# Enhanced effective connectivity from the middle frontal gyrus to the parietal lobe is associated with impaired mental rotation after total sleep deprivation: An electroencephalogram study

**DOI:** 10.3389/fnins.2022.910618

**Published:** 2022-09-30

**Authors:** Yutong Li, Mengke Ma, Yongcong Shao, Wei Wang

**Affiliations:** ^1^School of Psychology, Beijing Sport University, Beijing, China; ^2^Department of Criminal Psychology, Northwest University of Political Science and Law, Xi’an, China

**Keywords:** sleep deprivation, mental rotation, event-related potentials, effective connectivity, P300

## Abstract

Sleep deprivation impairs cognitive functions, including attention, memory, and decision-making. Studies on the neuro-electro-physiological mechanisms underlying total sleep deprivation (TSD) that impairs spatial cognition are limited. Based on electroencephalogram (EEG) and Exact Low Resolution Brain Electromagnetic Tomography (eLORETA), this study focused on the effects of TSD on mental rotation and the cognitive neural mechanisms underlying its damage. Twenty-four healthy college students completed mental rotation tasks while resting and after 36 h of TSD; their EEG data were simultaneously recorded. The amplitude of P300 component associated with mental rotation was observed and localized through source reconstruction, while changes in effective connectivity between multiple brain regions associated with mental rotation cognitive processing were calculated using isolated effective coherence (iCoh) of eLORETA. Compared with the baseline before TSD, the amplitude of the P300 component related to mental rotation decreased. The task-state data of P300 were localized to the source of the difference in ERP current density, and it was found that the brain regions related to the difference in the decrease in P300 amplitude included the superior parietal lobule, precuneus, prefrontal lobe, and other related regions. Effective connectivity analysis found that TSD enhanced the effective connectivity from the left middle frontal gyrus to the left superior parietal lobule, left inferior parietal lobule, and left precuneus under the identical condition. Pearson correlation analysis showed a positive correlation between the decrease in accuracy of mental rotation and increase in effective connectivity. Thus, our study suggests that TSD impairs the ability of the mental rotation, showing a decrease in P300 amplitude and an enhanced effective connectivity between the middle frontal gyrus and the parietal lobe in the task state.

## Introduction

Sleep loss is common in modern life. It not only causes various physical hazards, such as subjective fatigue and drowsiness, but also has a negative impact on cognitive function. Prolonged sleep deprivation can lead to decreased concentration (Doran et al., 2001; Shrimukhi et al., 2020), decreased alertness (Penetar et al., 1993), impaired memory (Cote et al., 2014), and decreased decision-making ability (Whitney et al., 2015); people may even make operational mistakes, leading to serious accidents (Mitler et al., 1988). Therefore, it is important to understand the mechanism of the influence of sleep deprivation on cognitive function for better physical and mental health.

Spatial cognition is one of the most basic human cognitive abilities (Schaie and Baltes, 1996). As a special visual-spatial cognitive task, mental rotation test can reflect an individual’s spatial cognition ability by imagining and recognizing a two-dimensional or three-dimensional object rotated at a certain angle from its original upright position (Cooper and Shepard, 1973; Riecansky and Jagla, 2008). Shepard et al. first discovered mental rotation, a classic psychological cognitive process, in 1971. Participants were asked to make equivalence judgments about a pile of three-dimensional figures in different orientations and determine whether the two images had the same relationship. Typical results showed that the response time of the judgment task increased linearly with the rotation angle (Shepard and Metzler, 1971). Completing the mental rotation task requires at least five cognitive steps (Booth et al., 2000): (I) visual perception and encoding of presented objects; (II) imagining of objects and their orientation; (III) mental rotation of objects; (IV) judging whether the object is similar to the target object; (V) making the final decision. The significance of studying mental rotation is to measure the subject’s visuospatial ability and study the process of spatial imagination and cognition (Carroll, 1993; Riecansky and Jagla, 2008; Höffler, 2010).

At present, there are very few studies on spatial cognitive functions during sleep deprivation, and research on mental rotation only remains at the behavioral level. Feng et al. (2018) conducted an experiment of 36 h of total sleep deprivation (TSD). Through a mental rotation task, they found that in the case of severe sleep loss, the spatial cognition of the participants decreased to a certain extent, which was reflected in decreased accuracy at the behavioral level. However, the neural mechanisms that underlie the effects of sleep deprivation on mental rotation remain unclear. In a special task that tests an individual’s spatial cognition ability, people with low cognitive ability had low mental rotation ability and long reaction times (Ma et al., 2018). Some studies have shown that sleep deprivation impairs individual cognitive function, which can provide a reference for studying mental rotation. García et al. (2021) conducted a 24-h sleep deprivation experiment and found that several specific components of basic cognitive processes (attention, working memory, and cognitive function) were significantly reduced after sleep deprivation. In addition, studies have demonstrated that TSD leads to progressive impairment of cognitive abilities, the most obvious of which is working memory (Lim and Dinges, 2010; Krause et al., 2017). The storage and manipulation of spatial representations in cognitive processing of mental rotation are closely related to the support of visual working memory. Just and Carpenter (1985) found that it was difficult for participants with poor visual working memory to maintain spatial information in spatial imagery tasks. Sleep deprivation has also been found to impair neurologic-related brain functions such as immediate memory (Thomas, 2003). Cote et al. (2014) suggested that sleep deprivation damages the hippocampus and may affect memory by disrupting synaptic plasticity. These research results on sleep deprivation effects on various cognitive functions can provide great reference value for studying how sleep deprivation affects the psychological rotation ability of spatial cognition.

Electroencephalography (EEG) has a high temporal resolution and is an effective tool for studying the neural mechanisms of cognitive function (Riečansky et al., 2013). Peronnet and Farah found in 1989 that in the mental rotation time window (350–800 ms), with the increase of rotation angle, the event-related potential (ERP) amplitude of the stimulus deviating from the upright direction gradually becomes more negative, which is known as rotation-related negativity (Stuss et al., 1983; Wijers et al., 1989). Subsequently, this negative ERP component was thought to be superimposed with the positive P300 ERP component and typically appeared 400 ms after stimulus presentation, most pronounced in the parietal lobe of the brain (Heil and Rolke, 2002). P300 is an endogenous ERP component that reflects the evaluation of stimuli (Polich, 2007) and allocation of attention (Stewart et al., 2010) during cognitive processing. Several ERP studies have found that sleep deprivation causes a decrease in the P300 amplitude (Morris et al., 1992; Panjwani et al., 2010). A decline in the P300 wave might account for decline in individual attention and recognition of stimuli (Koslowsky and Babkoff, 1992). It has also been demonstrated that mental rotation induces the activation of the frontal and parietal regions of the brain (Bhattacharya et al., 2001; Silberstein et al., 2003). Electrophysiological studies on the parietal cortex of monkeys have shown that the lateral intraparietal region within the internal parietal sulcus contains neurons that provide continuous and precise encoding of the object position relative to the observer (Gnadt and Andersen, 1988; Duhamel et al., 1992). Activation of the prefrontal cortex regions is thought to be involved in comparing target objects with mentally rotated objects (Carpenter et al., 1999), and activation of the parietal cortex regions is recognized to be involved in mediating visual and somatosensory inputs and generating visuospatial rotational movements (Podzebenko et al., 2005). These regions mediate the visuospatial transformations necessary for precise interactions with the environment (Stein, 1992; Milner and Goodale, 1995). Furthermore, a neural mechanism that correlates with intelligence and individual performance in mental rotation, could be a functional interaction between the frontal and parietal cortical regions, showing hemispheric differences in ERP amplitude in the frontal lobe but not in the parietal lobe (Anomal et al., 2020). Additionally, previous research has found that sleep deprivation disrupts activity in various brain regions. Shao et al. (2013) found that TSD leads to the disruption of thalamocortical functional connectivity, which may affect cognitive function. A functional magnetic resonance imaging (fMRI) study on primary insomnia found that TSD leads to altered functional connectivity in the parietal and the frontal lobes, which are important for spatial and verbal working memory. Although numerous electrophysiological and neuroimaging studies have investigated the effects of sleep deprivation on cognitive function (Doran et al., 2001; Cote et al., 2014; Mishra et al., 2020; García et al., 2021), studies on the effects of sleep deprivation on mental rotation using these techniques are lacking.

This study aimed to explore the effects of sleep deprivation on the performance of mental rotation tasks, using EEG and eLORETA technology. By observing changes in the ERP components and effective connectivity related to the mental rotation task before and after sleep deprivation, we focused on the effect of sleep deprivation on mental rotation and its cognitive neural mechanisms. The experiment used the classic mental rotation task of letters, replacing Shepard’s three-dimensional stimuli with letter stimuli, and the subjects were required to judge whether the two letters were in the same relationship (Shepard and Metzler, 1971). The present study documents the differences in behavioral data (response time and accuracy of mental rotation task), P300 amplitude component, and effective connectivity before and after TSD. We investigated changes in mental rotational ability following TSD using descriptive analysis and repeated measures analysis of variance (ANOVA). In addition, we performed a retrospective analysis of the P300 component of the mental rotation task and explored changes of inner connections between the brain regions associated with mental rotation after TSD. Based on previous findings, we hypothesize that (I) mental rotation ability decrease following sleep deprivation, as reflected in the prolonged response time and decreased accuracy of participants preforming mental rotation task, along with the decreased amplitude of the P300 component. However, this study illustrates the change of effective connectivity calculated using eLORETA, which is the innovation of this paper, so we hypothesize (II) following TSD, the interaction between brain regions associated with mental rotation is blocked, thereby altering the effective connectivity between brain networks, which is one reason for the decline of mental rotation ability.

## Materials and methods

### Participants

Thirty healthy men (mean age, 22; range, 21–25 years) participated in the study and all were right-handed with normal or corrected-to-normal vision. They had not participated in psychophysiology-related experiments before, and had undergone a rigorous physical examination to exclude mental and physical disorders. Their scores on the Pittsburgh Sleep Quality Index Questionnaire were less than 5 (Buysse et al., 1989), which indicated that they all had good sleep quality. Their intelligence quotient (IQ) was above the population average (IQ > 110). During the 2 weeks prior to experimentation, participants were instructed to report their daily sleep duration to ensure a regular daily sleep schedule of 7–9 h. Before the experiment, the main staff explained the experimental process and precautions in detail to the subjects. All participants filled out the informed consent form and received an experiment fee after the trial. The study was approved by Biological and Medical Ethics Committee of Beihang University and was performed in line with the principles of the Declaration of Helsinki.

### Mental rotation tasks

**FIGURE 1:**
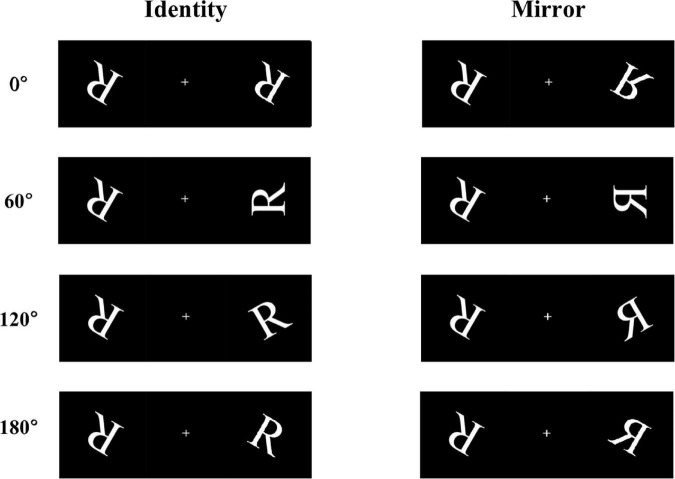
Some examples of the stimulus materials, including four angles of rotation (0°, 60°, 120°, and 180°), under two conditions (identical and mirror letters).

Participants were asked to perform a mental rotation task on an alphabetic character using a computer. The letter R was chosen as the stimulus material (Figure 1). The stimulus materials consisted of a pair of two letters R. The two letters were presented symmetrically on the left and right sides of the screen, which were either identical or mirror images of each other. There were four rotation angles in each form (0°, 60°, 120°, and 180°), which represented the angular disparities between the two letter stimuli “R.” Participants were required to judge whether the paired letter R had the same relationship; if so, they were to press the “F” key on the computer keyboard, if not, press the “J” key. During the experiment, participants were to respond using the keys as quickly as possible under the premise of making correct judgments.

The task lasted for approximately 20 min. At the beginning of the experiment, a fixation point “**+**” was presented at the center of the computer screen for a random duration (between 200 and 500 ms), and then the stimulus pictures were presented. Within 2 s, the participants were instructed to respond while making correct judgments. If the participants did not respond within 2 s, or the reaction lasted longer than 2 s, the stimulus automatically disappeared. Prior to the start of the formal experiment, all participants conducted practice tests and could proficiently operate the mental rotation tasks using the keyboard. In the formal experiment, each participant was given 512 trails in total; every condition of letter stimuli was shown in 256 trails, in which each angle (0°, 60°, 120°, and 180°) was randomly selected 64 times. In addition, the baseline and 36-h-TSD procedures were the same. Trials with response times shorter than 200 ms or longer than 2000 ms, as well as incorrect responses, were rejected.

### Experimental procedures

In the week prior to experimentation, participants were instructed to refrain from drinking alcohol, tea, coffee, or strenuous exercise, and strictly prohibit the use of various central nervous system inhibitory or stimulant drugs. The experiment adopted a one-group pretest-posttest design, and participants completed a 36 h TSD experiment. Participants arrived at the laboratory 2 days before the experiment, and the experimental procedures were explained and simulated to achieve a certain degree of familiarity with the experimental procedures and instruments. The formal experiment began after a night of normal sleep. The experiments were conducted in a dark, quiet, and electronically shielded EEG laboratory. To ensure normal work and rest, the participants slept in the laboratory for 2 days. At 8:00 in the morning of the formal experiment day, the participants were subjected to sleep deprivation, and the first mental rotation task was performed. EEG and behavioral data were collected. After 36 h of TSD (that is, at 20:00 the next day), the participants completed the second mental rotation task; the EEG and behavioral data were recorded at the same time. During the sleep deprivation period, participants were only given three meals a day at 7:30, 12:00, 17:30, respectively. All of the subjects spent most of their time sitting in their chairs and were allowed to stand up and walk slightly when they desired it. In principle, they could do anything as long as they did not nap, including playing games or watching movies, but strenuous physical activity and violent movies and games were forbidden and they could not leave the laboratory field as well. Three staff members (including one medical staff and two main personnel) supervised the participants to prevent them from sleeping (including naps).

### Statistical analysis of behavioral experiments

Behavioral data included reaction times and error rates, which were recorded and analyzed at the baseline and the 36-h TSD states. These analyses were performed using SPSS V22.2 (IBM Corporation, Armonk, NY, USA), including descriptive analysis and repeated measures analysis of variance (ANOVA). Within-subject repeated measures ANOVA was performed for reaction times and error rates using 2 (deprivation time: 0 h vs. 36 h) × 4 (angles: 0°, 60°, 120°, and 180°) factors. The Greenhouse-Geisser test was used to correct for cases that did not meet the sphericity test. In addition, Bonferroni correction was used for pairwise comparisons, and statistical results were expressed as mean and standard deviation (SD). If *p*_*FDR–corrected*_ was <0.05 (Benjamini and Hochberg FDR), the data were considered statistically significant.

### Electroencephalogram equipment and recordings

The mental rotation tasks were written and presented using the STIM 2 software (Neuroscan Inc., Charlotte, NC, USA). During the experiment, the participants sat on a soft chair approximately 75 cm away from the computer monitor (a 21-inch color monitor, 47.6 × 26.8 cm), and the center of their visual field was always flush with the center of the monitor. Based on the international standard 10–20 electrode placement system for sleep, the EEG data adopted Neuroscan Nuamps Amplifier (Scan 4.5, Neurosoft Labs Inc., Sterling, VA, USA) 64-lead EEG recording and analysis system, connected to the SynAmps2 amplifier at a sampling rate of 1000 Hz, and recorded the continuous scalp EEG. The impedance of all channels was maintained below 5 kΩ, and another four electrodes were placed on the upper and lower sides of the left and right eyes to record the vertical and horizontal electrooculograms.

### Electroencephalogram data analysis

The raw EEG signals were preprocessed offline using the EEGLAB 2020_0 toolbox in MATLAB R2020B (Mathworks, Natick, MA, USA). We merged the behavioral data then preprocessed the EEG data in the EEGLAB. Following EEG preview, we manually deleted an average of six trials with apparent artifactual signals and extracted epochs ranging from −200 to 800 ms of the continuous EEG data. The sampling rate was reduced to 250 Hz, while the average reference was performed as re-reference.

A band pass filter of 0.1–35 Hz was used *via* the Hamming windowed sinc FIR filter (transition band width = 2 Hz, order = 414) in the EEGLAB plugin, while the independent component analysis (ICA) was performed on the data. ICA can decompose the EEG data into independent signal, following which the ADJUST1.1.1 plugin was used to identify and remove the independent components that symbolize human eye artifacts and inordinate muscle activity. A total of nine independent components were removed on average from each subject data. The baseline was corrected in the range of −200 to 0 ms before the stimulus onset. Subsequently, the segments of each stimulus were averaged, and the average amplitude of P300 in the 300–500 ms period was measured. The Pz electrode was selected as an example for analysis.

All ERP results were analyzed using SPSS V22.2, and the mean amplitude of P300 waves was calculated using 2 (deprivation time: 0 h vs. 36 h) × 4 (angles: 0°, 60°, 120°, and 180°) within-subjects repeated measures ANOVA. In addition, Bonferroni correction was used for pairwise comparisons.

### Brain source localization analysis method

Exact Low Resolution Brain Electromagnetic Tomography (eLORETA; Pascual-Marqui et al., 2011) was used to localize the current density difference between the 300–500 ms ERP of the mental rotation task before and after sleep deprivation. eLORETA is an EEG analysis method for displaying the 3-dimensional image of the distribution of brain electrical activity sources. It allows the depiction of the source localization of the electric brain activity from scalp EEG data (Corsi-Cabrera et al., 2007; Pascual-Marqui et al., 2011; Cannon et al., 2012). The current source density analysis of eLORETA is based on a distinct, linear, weighted minimum norm inverse solution (Pascual-Marqui et al., 2011). In the eLORETA system, the analyzed space is limited to the cortical gray matter, separated by 6,239 voxels at 5 × 5 × 5 mm spatial resolution. As a head model of eLORETA, the Montreal Neurological Institute average MRI brain (MNI152) was used. According to Pascual-Marqui (2007), eLORETA is a genuine inverse solution that provides exact localization, with zero error, in the presence of measurement and structured biological noise. In addition, previous studies have shown that eLORETA can be used to locate deep brain structures, such as the subgenual anterior cingulate cortex (Pizzagalli et al., 2004) and medial temporal lobe with precision (Pascual-Marqui et al., 2014; Ridder et al., 2015) and have demonstrated the feasibility and reliability of the eLORETA method (Ikeda et al., 2015; Spyrou et al., 2016; Yuan et al., 2016).

### Isolated effective coherence

Since scalp signals cannot be used to calculate cortical connectivity, we used low-resolution electromagnetic tomography (eLORETA) to generate estimated cortical signals and calculate the strength of connectivity (Pascual-Marqui, 2007; Pascual-Marqui et al., 2011). Effective connectivity was calculated using the isolated effective coherence (iCoh) function of the eLORETA to assess the direct paths of intracortical casual information flow of the oscillatory activity. Brain regions associated with mental rotation were selected as regions of interest (ROI) (Table 1) and the effective connectivity between them and changes following TSD were analyzed.

**TABLE 1:** Montreal Neurological Institute (MNI) coordinates of the centroid voxel for the frontal, parietal, temporal, and occipital lobes.

Structure	Regions of interest (ROI)	Brodmann area	Side	X	Y	Z
Temporal lobe	L. posterior inferior temporal lobe	19/37	L	−42	−74	−4
Temporal lobe	R. posterior inferior temporal lobe	19/37	R	42	−74	−4
Occipital lobe	L. dorsal extrastriate visual areas	19/39	L	−22	−71	25
Occipital lobe	R. dorsal extrastriate visual areas	19/39	R	22	−71	25
Parietal lobe	L. Parietal ROI	7	L	−33	−55	35
Parietal lobe	R. Parietal ROI	39	R	33	−55	35
Parietal lobe	R. Precuneus (IPS)	7	R	28	−57	54
Parietal lobe	L. Precuneus (IPS)	7	L	−28	−57	54
Parietal lobe	R. Inferior Parietal Lobule	40	R	41	−39	43
Parietal lobe	L. Inferior Parietal Lobule	40	L	−41	−39	43
Parietal lobe	R. Precuneus (SPL)	7	R	24	−64	51
Parietal lobe	L. Precuneus (SPL)	7	L	−24	−64	51
Parietal lobe	R. Superior Parietal Lobule	7	R	19	−56	66
Parietal lobe	L. Superior Parietal Lobule	7	L	−19	−56	66
Frontal lobe	L. Middle Frontal Gyrus	6	L	−26	5	63
Frontal lobe	R. Middle Frontal Gyrus	6	R	26	5	63

Time series of the electric neuronal activity were estimated using eLORETA at 6,239 cortical voxels, from which 16 cortical ROI were included in the connectivity analyses. This procedure was applied to the EEGs recorded under resting and TSD conditions. iCoh was estimated for the 16-time series in all recordings (48 in total) using an autoregressive order *p* = 7, which corresponds to the median order for all EEG recordings based on Akaike’s AIC (Akaike, 1974). A statistical comparison between resting and TSD conditions was also conducted for each frequency, pair of ROIs, and direction of connection. During the *t* statistical analysis, the randomization Statistical Non-parametric mapping were performed (number of randomizations = 5,000), and the corrected critical thresholds and *p*-values were computed; then, the threshold [t] values representing the difference of effective connectivity of ROI were obtained. Finally, the results were read through the PlotFunc function.

In this study, iCoh algorithm based on a multivariate auto-regressive model was used to calculate the effective functional connectivity of the ROI. The formula for the iCoh calculation is as follows (Pascual-Marqui et al., 2014):


$${\text{k}}_{\text{i}\leftarrow \text{j}}(\text{ω})=\frac{{\left[{\text{s}}_{\varepsilon }\right]}_{\text{ii}}^{-1}|{\left[\text{A}(\text{ω})\right]}_{\text{ij}}{|}^{2}}{{\left[{\text{s}}_{\varepsilon }\right]}_{\text{ii}}^{1}|{\left[\text{A}(\text{ω})\right]}_{\text{ij}}{|}^{2}+{\left[{\text{s}}_{\varepsilon }\right]}_{\text{jj}}^{-1}|{\left[\text{A}(\text{ω})\right]}_{\text{jj}}{|}^{2}}$$

where ω is the frequency, which is 0,1… N_*T*_-1; K_*i*__←__j_ (ω) is the iCoh value of brain region from the jth to the ith, and its value ranges from 0 to 1; [A (ω)]_*ij*_ is the discrete Fourier transform matrix with a causal relationship with regions j and i; S_ε_ is the covariance matrix.

As shown in Table 1, brain regions related to mental rotation, such as the parietal, frontal, temporal, and occipital lobes, were selected as regions of interest.

## Results

### Behavioral performance

The EEG data of six participants were excluded for technical problems, hence the remaining 24 participants were included in the following analysis as the valid data. The descriptive statistics of the response time and error rate before and after sleep deprivation are presented in Tables 2, 3 (the individual data was also presented as box plots in the Supplementary material). Under two conditions of the letter stimuli (identity and mirror), a 2 (deprivation time: 0 h vs. 36 h) × 4 (angles: 0°, 60°, 120°, and 180°) ANOVA was used to analyze the response time and error rate, respectively (see Table 4 for the results). Under the condition of the identical letter stimulus, the main effects of sleep deprivation on response time [*F*_(1,23)_ = 8.73, *p*_*FDR–corrected*_ = 0.011, 1-β = 0.81] and error rate [*F*_(1,23)_ = 4.86, *p*_*FDR–corrected*_ = 0.038, 1-β = 0.56] were significant. Compared with the baseline, participants’ response time was prolonged after 36-h TSD (829.22 ± 37.95 ms), and the error rate was significantly increased (0.21 ± 0.02). In addition, the main effects of the rotation angle on reaction time and error rate were also significant [*F*_(1.479,34.020)_ = 84.99, *p*_*FDR–corrected*_ < 0.001, 1-β = 1.00; *F*_(1.896,43.604)_ = 76.48, *p*_*FDR–corrected*_ < 0.001, 1-β = 1.00]. The results of multiple comparisons showed that with the increase in the rotation angles of 0°, 60°, and 120°, the response time of the subjects was prolonged, and the error rate also increased, with statistical differences (*p* < 0.001). However, the response time for 180° rotation was not significantly different from that for 0° rotation; the error rate for 180° rotation (0.10 ± 0.02) was between 0° (0.05 ± 0.01) and 60° (0.20 ± 0.03), and participants had the highest error rate (0.43 ± 0.04) with an angle of 120°. In terms of the error rate, the interaction of sleep deprivation × angle was marginally not significant [*F*_(2.203,50.666)_ = 3.00, *p* = 0.054, 1-β = 0.58].

**TABLE 2:** Mean and standard deviation (SD) for the response time at baseline and TSD, separate for identical and mirror conditions.

	0°	60°	120°	180°
**Identity**				
Baseline-0h	586.60 ± 119.65	830.70 ± 117.07	969.79 ± 245.32	689.92 ± 132.06
TSD-36h	634.08 ± 156.94	891.65 ± 220.26	1058.13 ± 266.82	733.05 ± 165.45
**Mirror**				
Baseline-0h	676.55 ± 113.88	956.99 ± 217.41	1025.98 ± 280.05	756.36 ± 144.19
TSD-36h	704.21 ± 153.82	1010.54 ± 250.46	1070.01 ± 279.54	764.95 ± 185.35

**TABLE 3:** Mean and standard deviation (SD) for the error rate at baseline and TSD, separate for identical and mirror conditions.

	0°	60°	120°	180°
**Identity**				
Baseline-0h	0.03 ± 0.03	0.19 ± 0.13	0.39 ± 0.17	0.10 ± 0.89
TSD-36h	0.06 ± 0.07	0.22 ± 0.18	0.46 ± 0.20	0.10 ± 0.09
**Mirror**				
Baseline-0h	0.07 ± 0.07	0.28 ± 0.18	0.35 ± 0.19	0.10 ± 0.07
TSD-36h	0.07 ± 0.07	0.24 ± 0.17	0.33 ± 0.23	0.10 ± 0.10

**TABLE 4:** ANOVA statistic for behavioral (response time and error rate) and ERP data (Average amplitude of P300).

	Effect	Identity				Mirror			
		*F*(df)	*P*	η_*p*_^2^	1-β	*F*(df)	*P*	η_*p*_^2^	1-β
Response time	Sleep	*F*_(1,23)_ = 8.729	0.011*	0.275	0.808	*F*_(1,23)_ = 2.874	0.104	0.111	0.369
	Angle	*F*_(1.48,34.02)_ = 84.882	<0.001***	0.787	1.000	*F*_(1.44,33.08)_ = 80.550	<0.001***	0.778	1.000
	Sleep × angle	*F*_(1.66,38.20)_ = 1.217	0.301	0.050	0.231	*F*_(2.054,47.25)_ = 1.156	0.325	0.048	0.245
Error rate	Sleep	*F*_(1,23)_ = 4.862	0.038*	0.175	0.561	*F*_(1,23)_ = 0.991	0.330	0.041	0.159
	Angle	*F*_(1.90,43.60)_ = 76.475	<0.001***	0.769	1.000	*F*_(1.28,29.35)_ = 35.668	<0.001***	0.608	1.000
	Sleep × angle	*F*_(2.20,50.67)_ = 2.997	0.54	0.115	0.583	*F*_(2.00,46.01)_ = 0.720	0.492	0.030	0.164
Average amplitude of P300	Sleep	*F*_(1,23)_ = 6.566	0.021*	0.222	0.690	*F*_(1,23)_ = 6.506	0.020*	0.221	0.686
	Angle	*F*_(1.94,44.58)_ = 13.146	<0.001***	0.364	0.995	*F*_(2.00,46.10)_ = 4.998	0.015*	0.179	0.788
	Sleep × angle	*F*_(1.61,37.04)_ = 0.946	0.380	0.039	0.185	*F*_(2.071,47.64)_ = 0.525	0.601	0.022	0.133

df means degrees of freedom and * stands for *p* < 0.05, *** stands for *p* < 0.001.

Under the mirror condition, only the main effect of angle was significant, as reflected in both response time and error rate [*F*_(1.438,33.080)_ = 80.55, *p*_*FDR–corrected*_ < 0.001, 1-β = 1.00; *F*_(1.276,29.349)_ = 35.67, *p*_*FDR–corrected*_ < 0.001, 1-β = 1.00]. The results of multiple comparisons showed that with an increase in the rotation angles of 0°, 60°, and 120°, the participants’ response time was significantly prolonged, and the error rate was significantly increased, all of which were statistically significant (*p* < 0.001). However, the response time for 180°rotation was not significantly different from that for 0° rotation. The error rate for 180°rotation (0.10 ± 0.02) was between 0° (0.07 ± 0.01) and 60° (0.34 ± 0.04), and participants had the highest error rate (0.34 ± 0.04) at an angle of 120°.

### Amplitude

**FIGURE 2:**
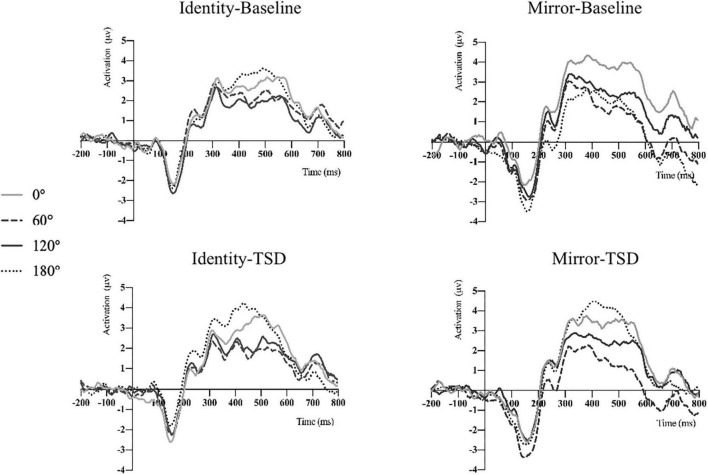
P300 average amplitude of mental rotation tasks before and after TSD was detected on the Pz electrode under the two conditions.

**FIGURE 3:**
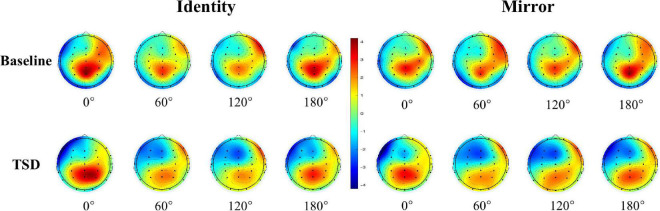
EEG topographic map of mental rotation tasks on the Pz electrode under different sleep conditions (baseline and TSD).

The descriptive statistics of the mean amplitude of the P300 waves in Pz electrode before and after sleep deprivation are presented in Table 5. Repeated measures ANOVA was performed on the mean P300 amplitudes under the two conditions for letter stimuli (see Table 4 for the results), and the ERP amplitudes are shown in Figure 2. The results showed that the main effect of sleep deprivation was significant in both identical [*F*_(1,23)_ = 6.57, *p*_*FDR–corrected*_ = 0.021, 1-β = 0.69] and mirror conditions [*F*_(1,23)_ = 6.51, *p*_*FDR–corrected*_ = 0.02, 1-β = 0.69]. Compared with the baseline, the mean amplitude of P300 decreased significantly after 36-h TSD, as reflected in the two conditions of the identical images (2.28 ± 0.41 μV) and mirror images (2.16 ± 0.45 μV). The main effect of the rotation angle was also significant for both conditions [*F*_(1.938,44.579)_ = 13.15, *p*_*FDR–corrected*_ < 0.001, 1-β = 1.00; *F*_(2.004,46.094)_ = 5.00, *p*_*FDR–corrected*_ = 0.015, 1-β = 0.79]. Under the identical condition, the results of multiple comparisons showed that the average amplitude of the P300 at a rotation angle of 0° was the largest (3.35 ± 0.39 μV), which was significantly higher than that at 60° (2.16 ± 0.37 μV) and 120° (2.17 ± 0.38 μV). There was no significant difference in the mean P300 amplitude between the rotation angles of 180° (2.99 ± 0.46 μV) and 0°. In addition, under the mirror condition, the rotation angle of 0° (2.93 ± 0.49 μV) had a larger P300 average amplitude than that of 120° (2.10 ± 0.41 μV) (*p* < 0.001). In addition, in the case of the two conditions, the most obvious distribution in the central and parietal lobes can be found both at the baseline and after the TSD of 36 h. The EEG topography is shown in Figure 3.

**TABLE 5:** Mean and standard deviation (SD) for the average amplitude of P300 at baseline and TSD, separate for identical and mirror conditions.

	0°	60°	120°	180°
**Identity**				
Baseline-0h	3.94 ± 2.49	2.43 ± 1.87	2.60 ± 2.10	3.25 ± 2.30
TSD-36h	2.76 ± 2.34	1.90 ± 2.09	1.74 ± 1.93	2.74 ± 2.48
**Mirror**				
Baseline-0h	3.17 ± 2.61	2.80 ± 1.96	2.49 ± 2.04	3.33 ± 2.23
TSD-36h	2.69 ± 2.52	1.93 ± 2.12	1.71 ± 2.20	2.33 ± 2.29

### Brain source localization analysis results

**FIGURE 4:**
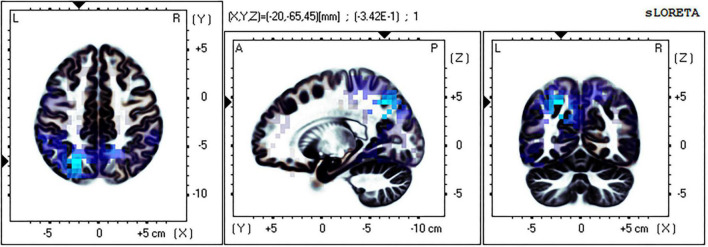
Brain source localization results of current density difference corresponding to 300–500 ms ERP data of mental rotation task after sleep deprivation and before sleep deprivation under the identical condition. Blue indicates the activation of these brain regions is significantly lower after TSD than before; Red indicates that the activation of these brain regions is significantly higher after TSD than before; from left to right: the axial, sagittal, and coronal planes, respectively. L, left; A, anterior; R, right; P, posterior.

**FIGURE 5:**
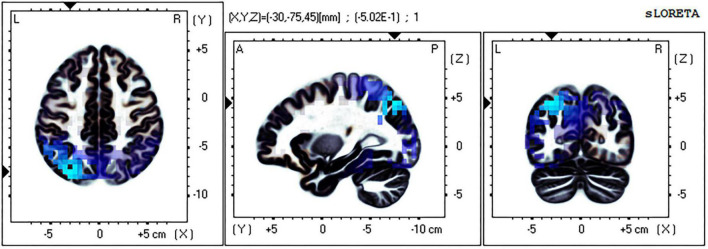
Brain source localization results of current density difference corresponding to 300–500 ms ERP data of mental rotation task after sleep deprivation and before sleep deprivation under the mirror condition. Blue indicates the activation of these brain regions is significantly lower after TSD than before; Red indicates that the activation of these brain regions is significantly higher after TSD than before; from left to right: the axial, sagittal, and coronal planes, respectively. L, left; A, anterior; R, right; P, posterior.

The eLORETA technology was used to localize the current density difference corresponding to the 300–500 ms ERP data of the mental rotation task after sleep deprivation and before sleep deprivation, and the results are shown in Figures 4, 5 (from left to right: the axial, sagittal, and coronal planes, respectively). Under the identical condition, the brain regions activated by processing differences before and after sleep deprivation, in the order of significance from large to small, were as follows: the superior parietal lobule (Brodmann area [BA]7), the precuneus of the parietal lobe (BA7, BA19), the superior temporal gyrus (BA22), the postcentral gyrus of the parietal lobe (BA43), the precentral gyrus of the frontal lobe (BA4, BA6), the angular gyrus of the parietal lobe (BA39), and the inferior frontal gyrus (BA45). Activation in these areas was significantly lower after sleep deprivation than before sleep deprivation. Under the mirror condition, the brain regions activated by processing differences before and after sleep deprivation, in the order of significance from large to small, were as follows: the superior parietal lobule (BA7), the precuneus of the parietal lobe (BA7, BA19, BA39), the angular gyrus of the parietal lobe (BA39), the inferior parietal lobule (BA7, BA39, BA40), the cuneus of the occipital lobe (BA19, BA18), the superior temporal gyrus (BA39), and the paracentral lobule of the frontal lobe (BA5). Activation in these areas was significantly lower after sleep deprivation than before sleep deprivation.

### Results of effective connectivity analysis

#### Effective connectivity changes under the identical condition

**FIGURE 6:**
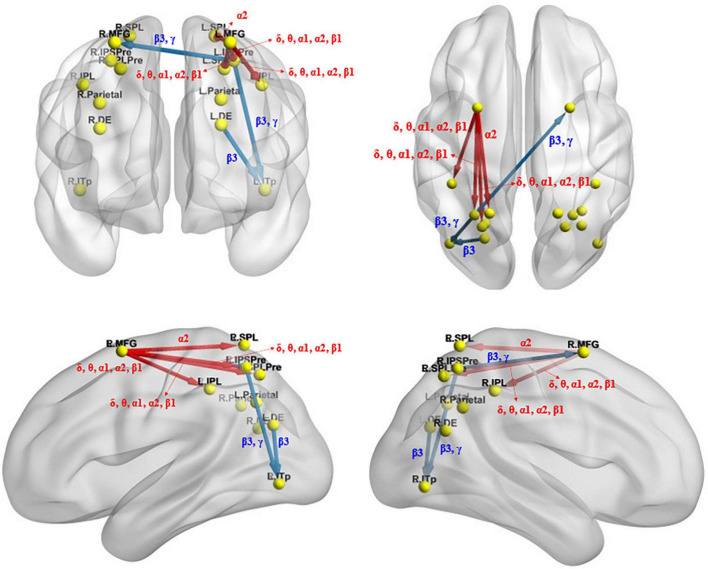
Changes in effective connectivity under the identical condition after total sleep deprivation. Compared with before sleep deprivation, the effective connectivity from the L.MFG to the L.IPSPre, L.IPL, L.SPLPre, and L.SPL were significantly enhanced. The effective connectivity from the L.DE to the L.ITp, from the L.IPSPre to the L.ITp and the R.MFG were significantly reduced. Blue indicates that the strength of the effective connectivity is smaller after total sleep deprivation than before; Red indicates that the strength of the effective connectivity is greater after total sleep deprivation than before. The arrow indicates the direction of the connectivity; From top to bottom and from left to right: coronal view **(frontal side)**, axial view **(dorsal side)**, sagittal view **(left side)**, sagittal view **(right side)**. The main statistically significant results are summarized. L.ITp, left posterior inferior temporal lobe; R.ITp, right posterior inferior temporal lobe; L.DE, left dorsal extrastriate visual areas; R.DE, right dorsal extrastriate visual areas; L.Parietal, left parietal ROI; R.Parietal, right parietal ROI; R.IPSPre, right precuneus [the intraparietal sulcus]; L.IPSPre, left precuneus [the intraparietal sulcus]; R.IPL, right inferior parietal lobule; L.IPL, left inferior parietal lobule; R.SPLPre, right precuneus [superior parietal lobule]; L.SPLPre, left precuneus [superior parietal lobule]; R.SPL, right superior parietal lobule; L.SPL, left superior parietal lobule; L.MFG, left middle frontal gyrus; R.MFG, right middle frontal gyrus. Frequency bands: δ, 1.5–6 Hz; θ, 6.5–8 Hz; α1, 8.5–10 Hz; α2, 10.5–12 Hz; β1, 12.5–18 Hz; β2, 18.5–21 Hz; β3, 21.5–30 Hz.

The iCoh analysis showed that, compared with before sleep deprivation, the effective connectivity between multiple brain regions was enhanced after sleep deprivation (*t*_95_ = 4.198, *p* = 0.004). This included the return of the left middle frontal gyrus to the left precuneus [the intraparietal sulcus] (*t*_95_ = 5.015, *p* < 0.004), left inferior parietal lobule (*t*_95_ = 4.627, *p* < 0.004), and left precuneus [superior parietal lobule] (*t*_95_ = 4.954, *p* < 0.004) in the δ, θ, α1, α2, and β1 bands, and the return of the left middle frontal gyrus to the left superior parietal lobule (*t*_95_ = 4.203, *p* < 0.004) in the α2 band. In other brain regions, the effective connectivity was reduced (*t*_95_ = 4.198, *p* = 0.004) after sleep deprivation, including the β3 band from the left dorsal extrastriate visual areas to the left posterior inferior temporal lobe (*t*_95_ = 4.328, *p* < 0.004), and β3 and γ bands from the left precuneus [the intraparietal sulcus] to the left posterior inferior temporal lobe (*t*_95_ = 4.443, *p* < 0.004) and the right middle frontal gyrus (*t*_95_ = 4.397, *p* < 0.004) (Figure 6).

#### Effective connectivity changes under the mirror condition

**FIGURE 7:**
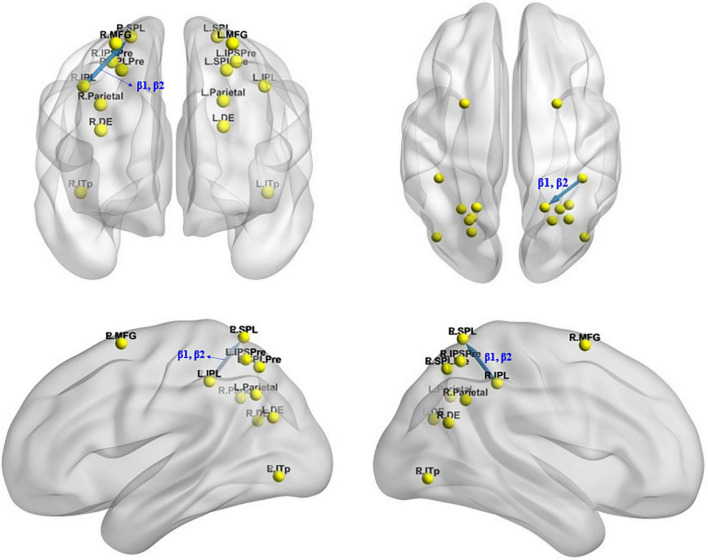
Changes in effective connectivity under the mirror condition after total sleep deprivation. The effective connectivity from the R.IPL to the R.SPL was significantly reduced compared with before sleep deprivation. Blue indicates that the strength of effective connectivity is smaller after total sleep deprivation than before; Red indicates that the strength of the effective connectivity is greater after total sleep deprivation than before. The arrow indicates the direction of the connectivity; From top to bottom and from left to right: coronal view **(frontal side)**, axial view **(dorsal side)**, sagittal view **(left side)**, sagittal view **(right side)**.

The iCoh analysis revealed that effective connectivity from the right inferior parietal lobule to the right superior parietal lobule was significantly reduced in β1 and β2 frequencies after sleep deprivation compared with before sleep deprivation (*t*_95_ = 4.247, *p* < 0.04) (Figure 7).

#### Correlation analysis between effective connectivity change and accuracy change

**FIGURE 8:**
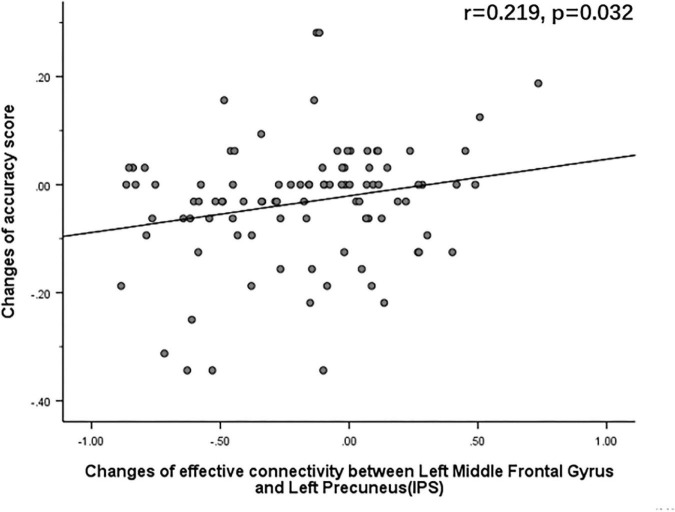
After sleep deprivation, there was a positive correlation, under the identical condition, between the change in effective connectivity from the left middle frontal gyrus to the left precuneus [the intraparietal sulcus] (*t*_95_ = 5.015, *p* < 0.004) and change in accuracy (*r* = 0.219, *p* = 0.032).

**FIGURE 9:**
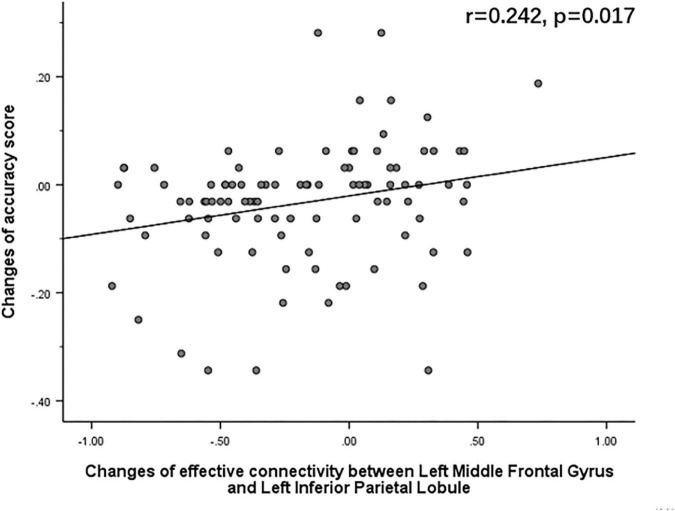
After sleep deprivation, there was a positive correlation, under the identical condition, between the change in effective connectivity from the left middle frontal gyrus to the left inferior parietal lobule (*t*_95_ = 4.627, *p* < 0.004) and change in accuracy (*r* = 0.242, *p* = 0.017).

**FIGURE 10:**
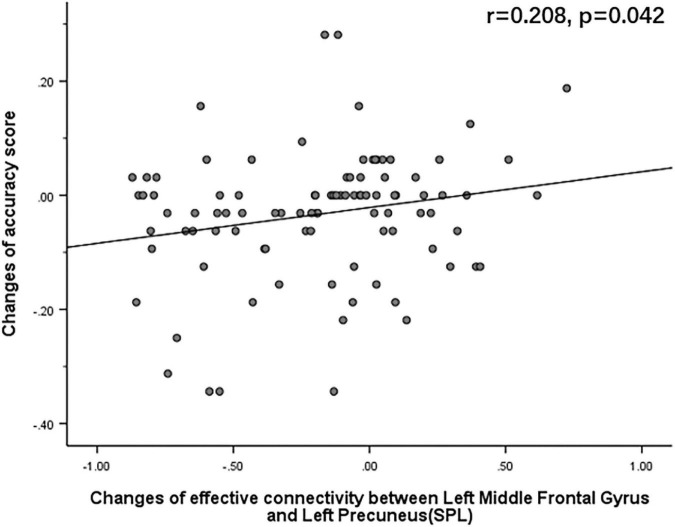
After sleep deprivation, there was a positive correlation, under the identical condition, between the change in effective connectivity from the left middle frontal gyrus to the left precuneus [superior parietal lobule] (*t*_95_ = 4.954, *p* < 0.004) and change in accuracy (*r* = 0.208, *p* = 0.042).

**FIGURE 11:**
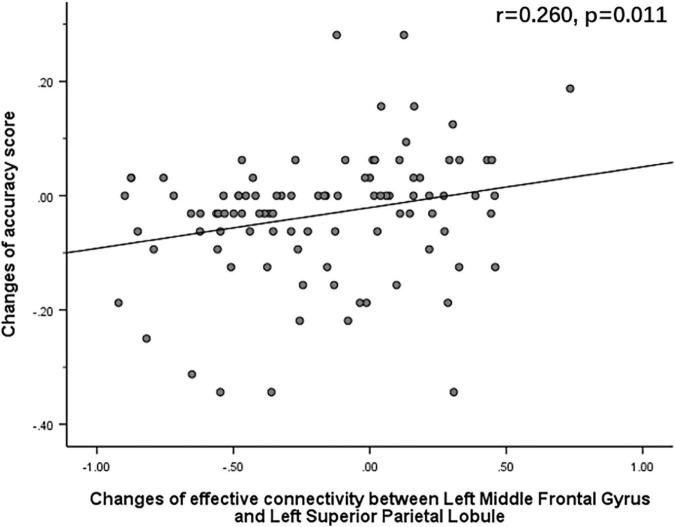
After sleep deprivation, there was a positive correlation, under the identical condition, between the change in effective connectivity from the left middle frontal gyrus to the left superior parietal lobule (*t*_95_ = 4.203, *p* < 0.004) and change in accuracy (*r* = 0.260, *p* = 0.011).

The Pearson correlation analysis showed that after sleep deprivation, there was a positive correlation under the identical condition between changes in accuracy and effective connectivity (Figures 8–11) from the left middle frontal gyrus to the left precuneus [the intraparietal sulcus] (*r* = 0.219, *p* = 0.032), to the left inferior parietal lobule (*r* = 0.242, *p* = 0.017), to the left precuneus [superior parietal lobule] (*r* = 0.208, *p* = 0.042), and to the left superior parietal lobule (*r* = 0.260, *p* = 0.011). However, our study did not find a significant correlation between changes in effective connectivity and changes in response time.

## Discussion

Using the ERP and task-state EEG technologies, we found that the mean amplitude of P300 component associated with mental rotation decreased significantly after sleep deprivation, which was consistent with our hypothesis (I). Brain source localization analysis using eLORETA showed that the brain regions associated with the amplitude drop difference in P300 included the parietal lobule, precuneus, and prefrontal lobe, and the activation of these regions was significantly lower after sleep deprivation than before. The iCoh analysis found that changes in the effective connectivity associated with sleep deprivation affecting mental rotational cognitive processing included enhancement of the effective connectivity from the left middle frontal gyrus to the left superior parietal lobule, left inferior parietal lobule, left precuneus [superior parietal lobule], and left precuneus [the intraparietal sulcus] under the identical condition and reduction of the effective connectivity from the left precuneus [the intraparietal sulcus] to left posterior inferior temporal lobe and right middle frontal gyrus, as well as reduction from left dorsal extrastriate visual areas to left posterior inferior temporal lobe. Additionally, the effective connectivity from the right inferior parietal lobule to the right superior parietal lobule was reduced under the mirror condition. These results of effective connectivity are also in line with the expectation of hypothesis (II). The Pearson’s correlation analysis also found that the increase in effective connectivity was positively correlated with the decrease in the accuracy following sleep deprivation, which confirms hypothesis (II). Studies have shown that increased effective connectivity from the middle frontal gyrus to the parietal lobe under the identical condition is associated with impaired mental rotation following total sleep deprivation.

Sleep deprivation impairs lower-order cognitive functions such as alertness and attention control (Killgore, 2010; Cunningham et al., 2018). Although individuals tried to perform mental rotation tasks in a state of wakefulness, according to the response time and accuracy of behavioral data, spatial cognitive ability was adversely affected and sleep deprivation affected the performance of the mental rotation tasks, which is in line with hypothesis (I). At the same time, it was found that between 0° and 120°, the response time of the subjects was prolonged, and the accuracy decreased with an increase in the rotation angle. When the rotation angle was 120°, the response time and error rate of the subjects reached a peak, which is consistent with previous research results (Cooper and Shepard, 1973; Bryden et al., 1990). An increase in the rotation angle means that the work of spatial representation becomes more difficult, and individuals will invest more psychological resources (Kessler and Thomson, 2009), resulting in longer processing time and lower accuracy of letter stimulus.

The P300 component is thought to reflect neurophysiological activities related to cognitively related processes such as attention and discrimination (Mitler et al., 1988; Lee et al., 2003). Sleep deprivation decreases the amplitude of the P300 component and prolongs latency (Morris et al., 1992; Panjwani et al., 2010; Ray et al., 2012). In this study, we measured the P300 component associated with the mental rotation task and observed a significant reduction in the mean amplitude of the P300 after complete sleep deprivation compared with the baseline at 0 h, which is in line with hypothesis (I). Studies have shown that sleep deprivation leads to a continuous decline in attention (Hudson et al., 2020). The average amplitude of P300 decreased significantly after sleep deprivation, indicating that individuals’ spatial cognition ability gradually collapsed, and sleep deprivation had an adverse effect on cognitive function, especially those dependent on psychology or attention (Goel et al., 2009; Killgore, 2010). The P300 component reflects individual responses to stimuli (Polich, 2007) and allocation of attentional resources (Stewart et al., 2010). In this study, we found that between 0° and 120°, the mean amplitude of P300 gradually decreased with an increase in the rotation angle, which is consistent with previous studies (Krause et al., 2021). The amplitude of the superposition of P300 and the rotation related negativity (RRN) is correlated with the rotation angle (Heil and Rolke, 2002). RRN usually occurs within 300–600 ms of stimulus presentation. As the rotation Angle difference increases, the amplitude of the positive component P300 becomes more negative due to the superimposed RRN (Ter Horst et al., 2012; Krause et al., 2021), which is thought to reflect the actual cognitive process of mental rotation (Peronnet and Farah, 1989). Moreover, Johnson et al. (1987) have suggested that larger P300 amplitude reflects an increase in individual stimulus perception.

Previous studies have shown that decreased brain activation in the frontal and parietal lobes after sleep deprivation occurs in a variety of cognitive tasks, including attention (Chee et al., 2010; Lim et al., 2010), memory encoding and consolidation (Fischer et al., 2005; Yoo et al., 2007) and speech learning (Drummond et al., 2000). However, research on mental rotation has been insufficient. The results of this study are generally consistent with those in other cognitive areas. The results of localizing the current density of the P300 component corresponding to the mental rotation task of both the identical and mirror conditions showed that the activation degrees of related regions, such as the parietal lobule, precuneus, temporal lobe, and prefrontal lobe, were significantly reduced after sleep deprivation compared with before sleep deprivation. All of these brain regions are closely associated with mental rotation. The precuneus is closely connected to several brain regions and plays an important role in memory retrieval (Cavanna and Trimble, 2006) and information collection (Wang et al., 2017). The precuneus belongs to the default network and plays a key role in maintaining alertness (Liu et al., 2015). The decreased activation of the precuneus leads to a decline in individual alertness, interferes with the working memory process to some extent, and damages individual mental rotation ability. Harris et al. (2000) conducted a mental rotation experiment and found significant activation centered on the internal parietal sulcus (BA7), which is involved in various visuospatial transformations. A study of patients with brain injury found that lesions in the right parietal lobe impaired normal mental rotation (Ditunno and Mann, 1990). The above studies have shown that the parietal lobe plays an important role in mental rotation, and the reduction of activation of parietal lobe areas will seriously damage the spatial cognitive ability of individuals, and thus the mental rotation ability of individuals, which is also demonstrated in this study. The middle temporal gyrus (MT/V5) and the medial superior temporal gyrus (MST) contain directionally selective cells capable of detecting mobile stimuli (Dubner and Zeki, 1971; Desimone and Ungerleider, 1986). In an fMRI study (Cohen et al., 1996) of mental rotation, activation of the frontal lobe was thought to represent the control of oculomotor function to scan two stimuli and the processing needs of working memory. Decreased activation in the temporal and frontal lobes may represent decreased visuospatial information gathering and processing abilities after sleep deprivation. In conclusion, sleep deprivation inhibits spatial cognition, working memory, and other abilities, leading to poor performance in mental rotation tasks.

In the mental rotation task, TSD not only reduced the effective connectivity between some brain regions associated with mental rotation, which is consistent with hypothesis (II) that information interaction between brain regions associated with mental rotation is blocked, but also enhanced the effective connectivity between the middle frontal gyrus and parietal lobe under the identical condition. These enhanced effective connections may compensate for the disruption in information communication following sleep deprivation, which are also associated with impaired mental rotation ability. Effective connectivity analysis of ERP data of mental rotation task showed that sleep deprivation reduced the effective connectivity from left dorsal extrastriate visual areas to left posterior inferior temporal lobe, from left precuneus [the intraparietal sulcus] to left posterior inferior temporal lobe and right middle frontal gyrus under the identical condition, and the effective connectivity from the right inferior parietal lobule to the right superior parietal lobule under the mirror condition. However, it increased the effective connectivity from the left middle frontal gyrus to left precuneus [the intraparietal sulcus], left inferior parietal lobule, left precuneus [superior parietal lobule], and left superior parietal lobule under the identical condition. The former reflects the impairment of spatial cognition caused by sleep deprivation, whereas the latter is more likely a compensatory strategy adopted by the brain to compensate for cognitive decline. Previous studies have shown that, after sleep deprivation, a compensatory mechanism related to the frontal and parietal lobes exists. An fMRI study on sleep deprivation conducted by Drummond et al. (2000) found that the frontal lobe was more sensitive after sleep deprivation than after normal sleep, and increased subjective sleepiness was significantly associated with activation of the frontal lobe. The parietal lobe was not activated after normal sleep but was activated after sleep deprivation, and better free recall in sleep-deprived subjects was associated with greater parietal lobe activation. The increase in blood oxygenation level dependent (BOLD) signal in the bilateral working memory areas of the prefrontal and parietal lobes caused by sleep deprivation during speech learning is thought to represent the neurophysiological mechanism of initial compensation by sleep deprivation. Chee and Choo (2004) suggested that increased prefrontal and thalamic activation after sleep deprivation might represent compensatory adaptation. A sleep deprivation experiment by Zhang et al. (2021) found an increase in effective connectivity between the left ventrolateral prefrontal cortex and left dorsolateral prefrontal cortex under matching conditions for the 2-back task. Lei et al. (2015) found that after sleep deprivation, the functional connectivity between the default-mode network (DMN) and salience network (SN) increased significantly, and the connection strength was correlated with sleepiness and working memory task response time. In this study, TSD enhanced the effective connectivity between the middle frontal gyrus and parietal lobe during the mental rotation task under the identical condition, suggesting that the brain prioritizes the investment of more energy to maintain information exchange between the middle frontal gyrus and parietal lobule under the state of excessive mental fatigue. The precuneus is the connecting link between the parietal and occipital lobes, and the enhancement of its effective connectivity may be related to the promotion of visual processing. The enhancement of these effective functional connectivity may be a compensatory mechanism used by the brain to address the negative effects of sleep deprivation on spatial cognition.

Pearson correlation analysis showed a positive correlation between the increase in effective connectivity from the left middle frontal gyrus to the left precuneus [the intraparietal sulcus], left inferior parietal lobule, left precuneus [superior parietal lobule], and left superior parietal lobule after sleep deprivation and the accuracy of mental rotation task under the identical condition. The results suggest that the enhancement of these effective connectivity is closely associated with the impairment of mental rotation ability and is strong evidence supporting hypothesis (II). The decrease in accuracy reflected that sleep deprivation impaired spatial cognition, whereas the enhancement of effective connectivity between the middle frontal gyrus and parietal lobe suggested that this was more likely a compensatory mechanism.

In conclusion, as we expect from hypothesis (I) and hypothesis (II),TSD did damage the spatial cognitive ability of individuals, which was manifested in the prolonged response time and decreased accuracy rate after TSD, decreased P300 amplitude, decreased activation of the parietal lobe, prefrontal lobe, temporal lobe, and other related brain regions, as well as the reduction of effective connectivity from left dorsal extrastriate visual areas to left posterior inferior temporal lobe, from left precuneus [the intraparietal sulcus] to left posterior inferior temporal lobe and the right middle frontal gyrus under the identical condition, and from right inferior parietal lobule to right superior parietal lobule under the mirror condition. However, we observed a compensatory mechanism underlying cognitive function following TSD, specifically, under the identical condition, the effective connectivity from the left middle frontal gyrus to left precuneus [the intraparietal sulcus], left inferior parietal lobule, left precuneus [superior parietal lobule], and left superior parietal lobule was enhanced. Moreover, the enhancement of these effective connectivity positively correlated with the decrease in accuracy of the mental rotation task. Therefore, we prefer the compensatory view.

However, our study had some limitations. First, we investigated how sleep deprivation affects object rotation using only letter stimuli in mental rotation task. Previous studies exploring the differences between using letters and figures [e.g., cube figures (Metzler and Shepard, 1974), human body parts (Amorim et al., 2006), and whole human bodies (Krause et al., 2021)] found that the difference of stimuli have important effects on both behavior (response time and accuracy) and neural data in mental rotation experiments. In the future, we intend to compare mental rotation tasks of body parts with those of objects to better understand how sleep deprivation affects mental rotation from a cognitive perspective. Second, participants experienced drowsiness during TSD, but the effects of the drowsiness and TSD on the EEG activity could not be distinguished; hence we intend to include a control group in future studies to distinguish the effects of TSD from drowsiness in mental rotation tasks. Third, mental rotation is a complex cognitive task that has several different stages, including the early cognitive stage, the late cognitive stage, and the mental rotation itself (Shepard and Cooper, 1982). We only discussed the mental rotation stage where the P300 component is present, and the cognitive neural mechanism caused by other stages is not clear. Fourth, the use of ICA as a method for artifact rejection and reconstructing the signal after removing the artifactual components can distort the content of the phase information between signals. Effective connectivity results can be altered by this ICA procedure. Fifth, due to the small sample size and the large impact of total sleep deprivation, we did not conduct a rigorous cleaning procedure for the behavioral data, which may therefore not be representative; in addition, only male volunteers were used in this study. Thus, our findings need to be validated in a larger sample including female subjects. Sixth, considering that it is still under investigation whether ICA artifact removal distorts phase, researchers need to carefully consider using ICA for artifact removal in the design of source reconstruction analysis in future studies. Finally, we cannot completely rule out the influence of circadian rhythms on test results. As previous studies have found that circadian rhythms affect people’s performance (Montplaisir, 1981; Lavie, 2001), we did not record EEG data at the same time point in the experiment.

In this study, the average amplitude of the P300 component in the mental rotation stage decreased under the influence of sleep deprivation, and mental rotation ability was impaired after TSD, which may be related to the reduction of spatial cognition and working memory by TSD. Sleep deprivation alters the effective connectivity associated with mental rotation, and TSD enhances the effective connectivity between the middle frontal gyrus and the parietal lobe, which may be related to the impairment of mental rotation after total sleep deprivation. This study provides electrophysiological evidence to understand the cognitive neural mechanisms underlying mental rotation impairment after sleep deprivation. It is necessary to pay attention to the adverse effects of sleep deprivation on mental rotation impairment in the future and to actively explore effective intervention measures.

## Data availability statement

The datasets generated for this study are available on request to the corresponding authors.

## Ethics statement

The studies involving human participants were reviewed and approved by the Ethics Committee of Beihang University. The patients/participants provided their written informed consent to participate in this study.

## Author contributions

YS designed the experiments. YL and MM produced the results and wrote the manuscript. MM and YL analyzed and interpreted the data. WW and YS performed the experiments, acquainted the data, and the guaranteed the study. YL, MM, and WW contributed to participating in data collection and reviewing the literature. All authors listed have read and approved the final manuscript.
