# Supplementary figures and images for: Enhanced effective connectivity from the middle frontal gyrus to the parietal lobe is associated with impaired mental rotation after total sleep deprivation: An electroencephalogram study
Source: Front Neurosci. 2022 Sep 30;16:910618. doi: 10.3389/fnins.2022.910618 (PMC9566834; doi:10.3389/fnins.2022.910618)

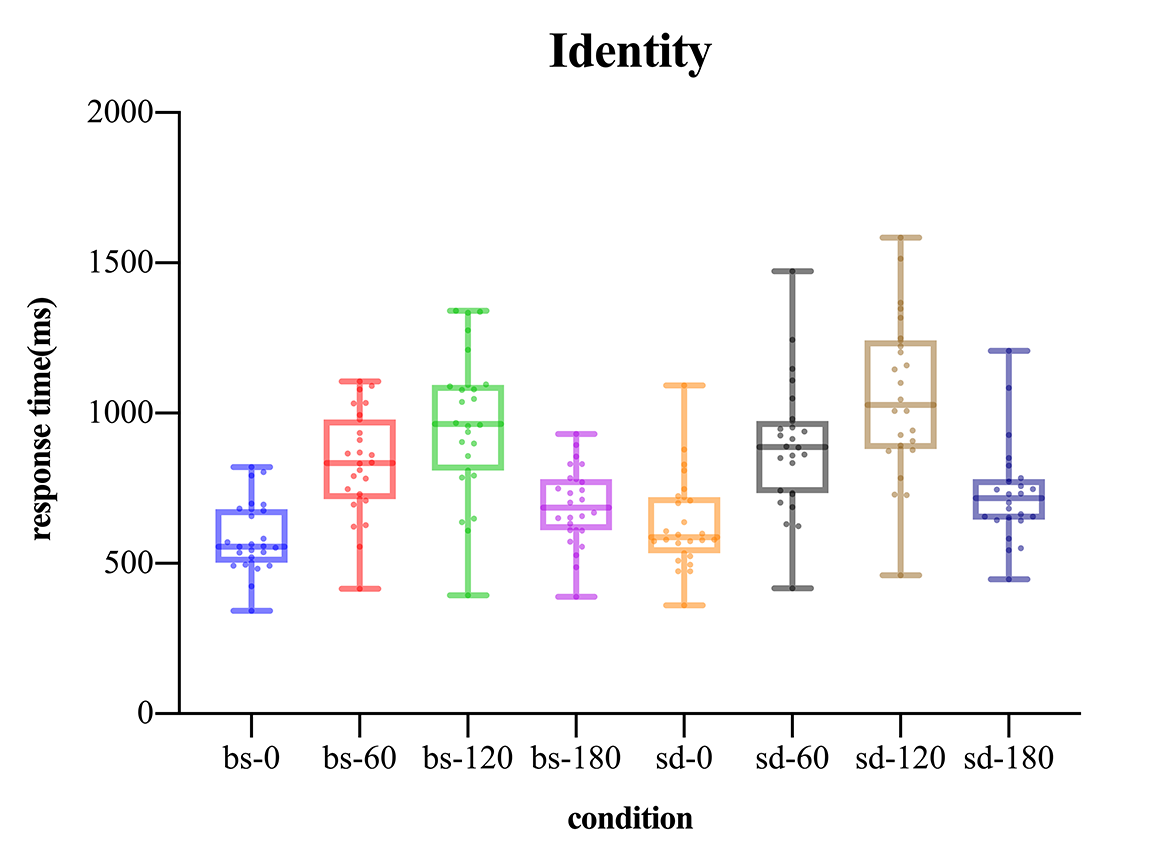

Supplement: Supplementary file 1 [file Image_1.TIFF]

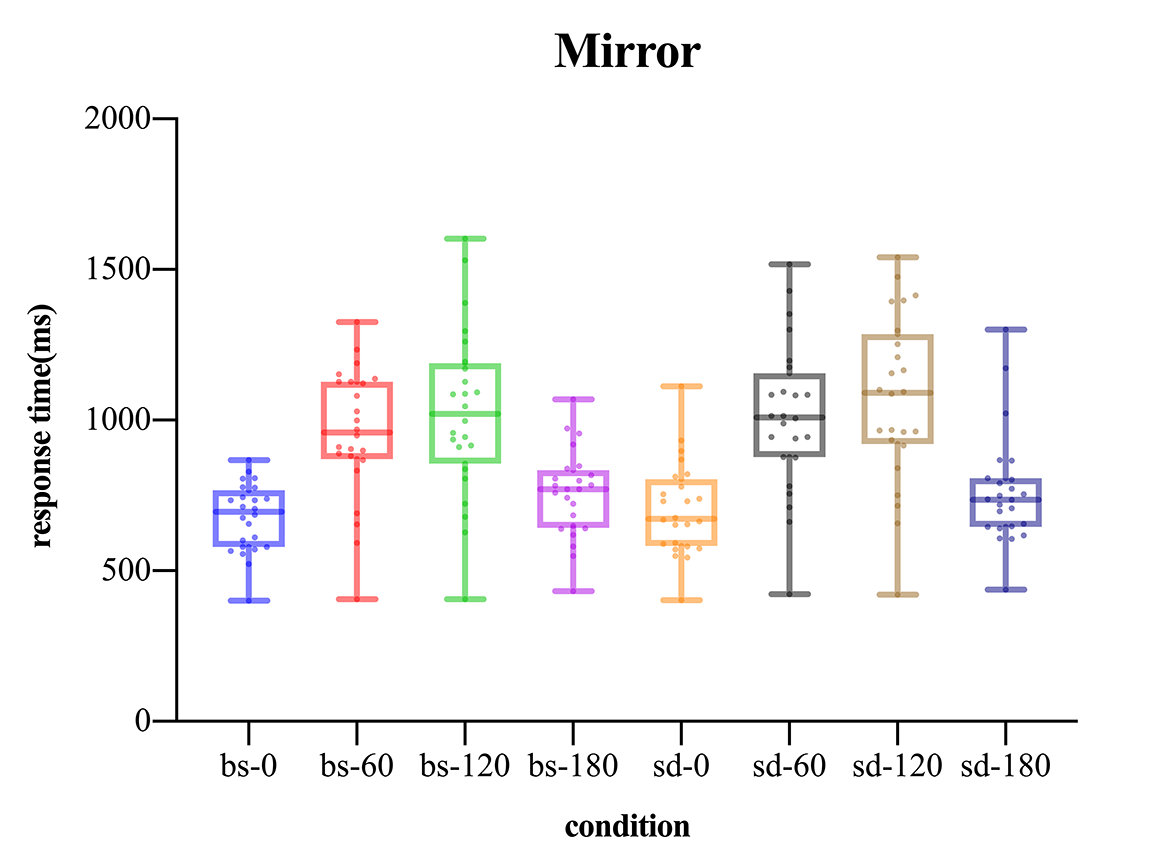

Supplement: Supplementary file 2 [file Image_2.TIFF]
